# Topology of Transcriptional Regulatory Networks: Testing and Improving

**DOI:** 10.1371/journal.pone.0040082

**Published:** 2012-07-23

**Authors:** Dicle Hasdemir, Gertien J. Smits, Johan A. Westerhuis, Age K. Smilde

**Affiliations:** 1 Biosystems Data Analysis, Swammerdam Institute for Life Sciences, University of Amsterdam, Amsterdam, The Netherlands; 2 Molecular Biology and Microbial Food Safety, Swammerdam Institute for Life Sciences, Amsterdam, University of Amsterdam, The Netherlands; 3 Netherlands Metabolomics Centre, Leiden, The Netherlands; University College London, United Kingdom

## Abstract

With the increasing amount and complexity of data generated in biological experiments it is becoming necessary to enhance the performance and applicability of existing statistical data analysis methods. This enhancement is needed for the hidden biological information to be better resolved and better interpreted. Towards that aim, systematic incorporation of prior information in biological data analysis has been a challenging problem for systems biology. Several methods have been proposed to integrate data from different levels of information most notably from metabolomics, transcriptomics and proteomics and thus enhance biological interpretation. However, in order not to be misled by the dominance of incorrect prior information in the analysis, being able to discriminate between competing prior information is required. In this study, we show that discrimination between topological information in competing transcriptional regulatory network models is possible solely based on experimental data. We use network topology dependent decomposition of synthetic gene expression data to introduce both local and global discriminating measures. The measures indicate how well the gene expression data can be explained under the constraints of the model network topology and how much each regulatory connection in the model refuses to be constrained. Application of the method to the cell cycle regulatory network of *Saccharomyces cerevisiae* leads to the prediction of novel regulatory interactions, improving the information content of the hypothesized network model.

## Introduction

In recent years, multiplex and high-throughput technologies provided biologists with the opportunity to increase the amount of data generated on various biological systems. Analysis of these data allows to gain comprehensive information on the system on various levels such as transcriptome, proteome, metabolome and interactome. However, there are two major challenges which are directed by the systems biology perspective. The first challenge is to integrate all the information from these different levels. Statistical approaches for this aim have mostly stemmed from the need of integrating transcriptome data with other omics data sets. A noticeable example in this field has focused on mapping gene expression data on protein-protein and protein-DNA interactome data to reveal the active sub-networks in the course of perturbation experiments [1].

The second challenge is to interpret the massive information collected from experiments in a biologically meaningful manner. Data analysis can be directed towards knowledge already available on the investigated system to facilitate its biological interpretation. This approach is referred to as incorporation of prior information in data analysis.

Systematic incorporation of prior information in data analysis has been an important topic in statistics mostly due to Bayesian approaches. With the increasing demand of statistical approaches in biology, methods have been proposed also in this particular area. Several studies have focused on exploiting different kinds of prior information in biological systems. One important approach is based on Factor Analysis directed by prior information. Network Component Analysis (NCA) [2] set the framework for the decomposition of microarray data based on the transcriptional regulatory network topology provided as prior information. This decomposition leads to the reconstruction of both the connection strengths between gene - transcription factor pairs and the transcription factor activities over a range of different conditions. The NCA approach has been the subject of several followup studies which aimed either at increasing the applicability range of the method [3], [4], the stability of the solutions [3] or finding more efficient ways of carrying out the decomposition involved [5].

In some studies, the prior information is exploited in a controlled manner where the analyst can set the limit for the intervention level of the prior information [6]–[9]. In these studies, penalized or stepwise regression methods and Bayesian approaches are utilized. By some of these approaches, the prior information is also changed in accordance with the data at hand [8], [9]. In [8] this is accomplished by updating the prior information back and forth between different prior information with different reliabilities whereas in [9] forward stepwise regression is used to determine the true positive interactions in the prior information.

However, there is one point which must always be kept in mind while incorporating any type of prior information in data analysis. It is very likely to lead to incorrect results if incorrect prior information is allowed to dominate the data analysis process. Therefore, being able to discriminate between competing sources of prior information has always been an important issue. With this study, we propose measures to make this discrimination available on both global and local levels based on the assumption that correct models must behave consistent with experimental observations. To explain it more clearly; if there are two different hypotheses for a certain type of prior information, these measures will guide us to identify which hypothesis (on global level) or which parts of each hypothesis (on local level) are supported more by the experimental data and thus are closer to the underlying biological reality. Our focus is on topological prior information in transcriptional regulatory networks. However, such an approach can also be used for discriminating between competing prior information at other levels such as metabolomics and proteomics when appropriately adapted.

In this paper, we show how two different regulatory networks can be distinguished on a global level using an NCA type decomposition framework. New regulatory interactions between genes and transcription factors can also be proposed by using our method. This feature represents our method’s local performance. Furthermore, we show how application of our method to cell cycle transcriptional regulatory network of *Saccharomyces cerevisiae* led to the improvement of the regulatory interactions in the network.

## Methods

### Guideline for Data Decomposition

A Factor Analysis model for gene expression data can be written as in Equation 1. This type of model relates the gene expression data to the underlying hidden factors, namely the activity of the transcription factors. In this decomposition scheme, **X** contains gene expression profiles of the I genes in the J conditions in terms of 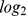 ratios. The score matrix **T** contains the binding association information between the I genes and the K transcription factors. The **P** matrix contains the activities of the K transcription factors in the J conditions in its columns. The matrix **E** contains the residual of the model, namely the part of the data that could not be modeled. Network Component Analysis (NCA) puts restrictions on the decomposition. In an NCA model, the score matrix **T** must be an element of Z, a special set of matrices. These matrices have a predefined structure based on the imposed topological pattern of the network. Binding of a transcription factor on the promoter region of a gene is represented with a nonzero value -the connection strength between the genes and the transcription factors- and lack of binding is represented with a 0. The decomposition in Equation 1 was proven to be unique up to scaling under certain criteria for the *identifiability* of the system [2]. The estimation of **T** and **P** under the imposed topological constraints gives us both the connection strengths between gene - transcription factor pairs as well as the transcription factor activities over a range of different conditions.
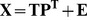
(1)


In our approach, the decomposition in Equation 1 is carried out by Alternating Least Squares with two types of constraints; topological constraints on **T** as NCA puts and unit column length constraint on **P**. In other words, as demanded by the first constraint, **T** has to stay a member of the set Z, the set of all the matrices which obeys the imposed topological pattern. The imposed topological pattern is represented by fixed places of zeros in **T**. With the second constraint, the length of all the columns in the estimated **P** (the activity profiles of all the transcription factors in the system) are fixed to unit length. This makes the comparison between different estimates of the **T** possible in different simulations as will be explained later.

**Figure 1:**
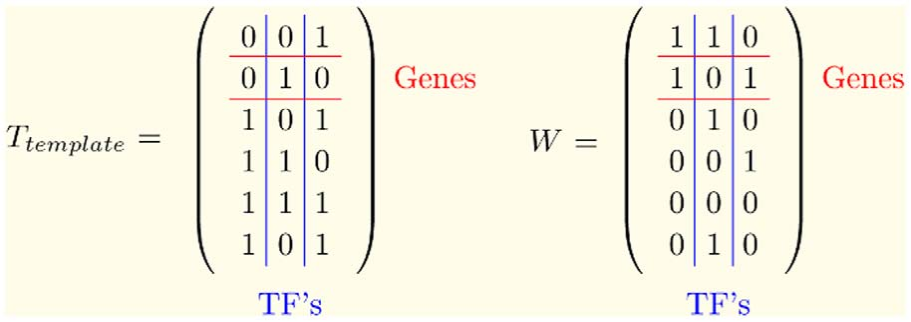
Example network with 6 genes and 3 transcription factors (TF’s). The imposed network structure is encoded in 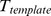. Existing connections are depicted as 1′s. W has 1′s at the positions where the difference between the initial estimate, 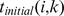 and the template, 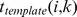 is subject to minimization. These positions correspond to the 0′s in 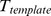 where no connections exist.



The **first step** of Alternating Least Squares is the initialization step. In this step, 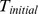, an initial educated guess for **T** is given. For obtaining this initial guess, a PCA decomposition is carried out on the data matrix **X**. The resulting score matrix, 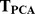 is a good initialization for **T** itself. However, the PCA score matrix can be rotated further towards the imposed topological pattern with the requirement of staying within the PCA space. So, the elements which are restricted to 0 based on the topology would be as close to 0 as possible and thus the nonzero elements would be adjusted accordingly. This is achieved by multiplying the PCA score matrix, 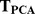 by a nonsingular rotating matrix, 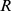. The minimization function for the estimation of 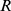 is given in Equation 2. The target minimization in Equation 2 is carried out only on the restricted elements as imposed by the binary matrix 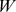 and the use of the Hadamard Product (Figure 1). The 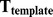 is the matrix in which the imposed topology is encoded with 1′s showing the interaction and 0′s showing lack of interaction between genes and transcription factors.

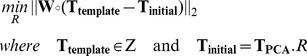
(2)In the **second step**, estimation of **P** is achieved by an Iterative Restricted Least Squares approach (Personal communication with Henk Kiers, University of Groningen) using the educated guess 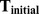. In the **third step**, Ordinary Least Squares is used for estimating **T** based on 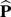 which was estimated in Step 2. In this step only the nonzero values in **T** are subject to change. The elements which are restricted to 0 as imposed by the network topology are always kept as 0. The computation in this step follows the guideline which was defined within the NCA framework [2]. The constraints and objectives of the overall optimization scheme are summarized in Equation 3 where the estimated variables are shown with a hat 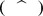 on them. The **fourth step** is the termination step where the alternating least squares algorithm is terminated when the relative change in the residuals is below a previously determined threshold.

**Figure 2:**
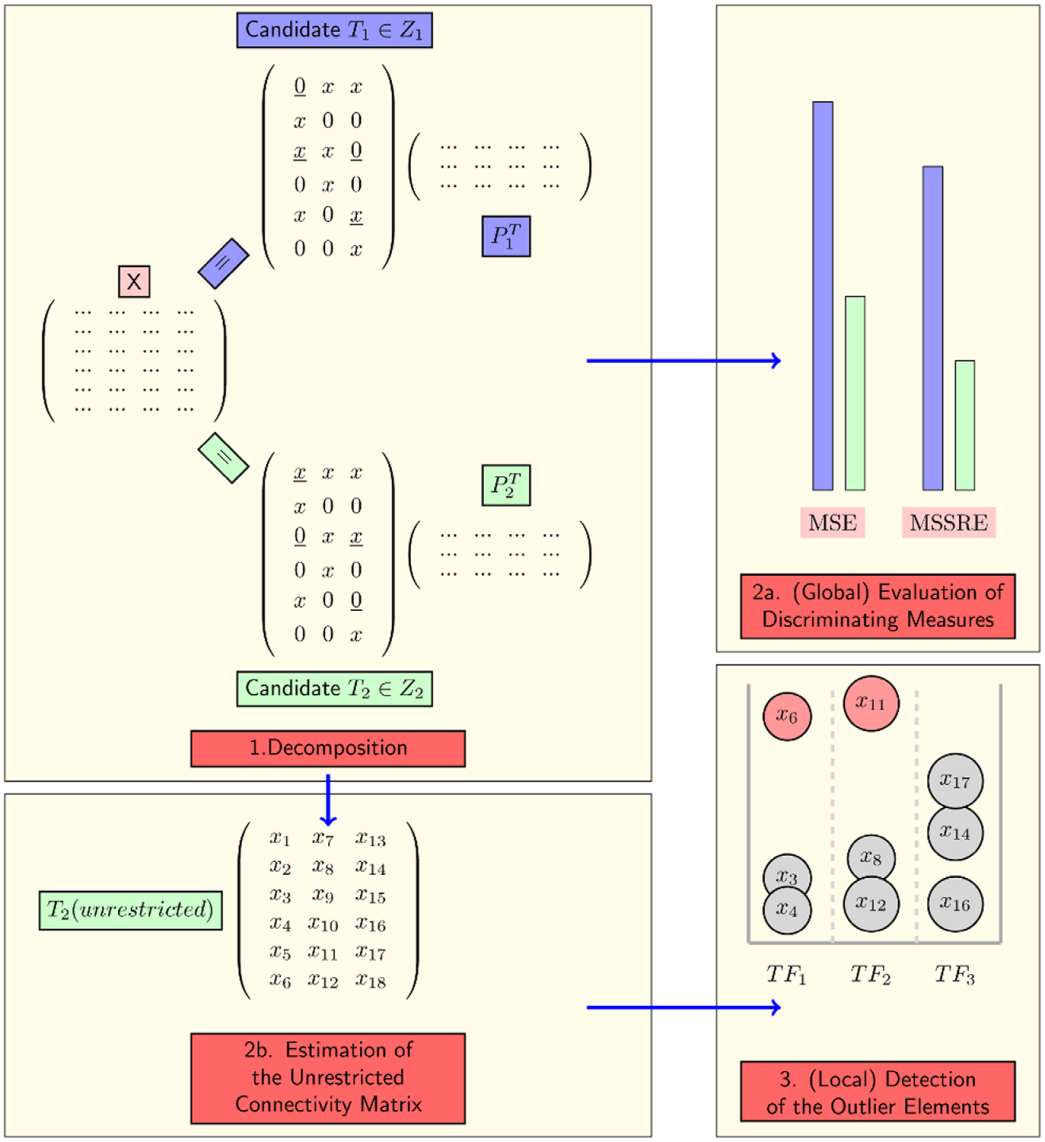
General view of the approach. The individual steps in the figure are explained in detail in the Methods Section.

How this type of supervised decomposition of gene expression data is used to provide us with a guideline to discriminate between competing network information is depicted in Figure 2.


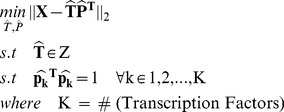
(3)

### Discriminating Measures

#### MSE: Mean sum of squared residuals in the model

The first proposed measure is the model fit. This simple but yet very important measure is strongly dependent on the prior information regarding the network structure. The concept of model fit as a discriminating measure is based on the idea that the model data matrix, 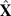 will be closer to the measured data matrix, **X** when a network model which is closer to the real network structure is used as prior information. This is due to the strictness of the constraints imposed by the topology of the network.

MSE is calculated via Equation 4 as the mean sum of squared residuals in the model.
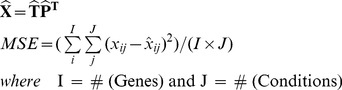
(4)


#### MSSRE: Mean squared sum of restricted elements

The second proposed measure uses 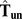 which is the connectivity matrix estimated after the relaxation of the topological constraints. MSSRE summarizes the distance of specific elements from 0 in 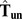. These specific elements are the ones which were restricted to 0 in the imposed network structure.

Once the alternating least squares is terminated and the 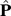 matrix is estimated, we can obtain the unrestricted connectivity matrix, 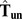 by solving Equation 5. This would be the ordinary least squares solution to the problem under no topological constraints. So, there are now two predicted connectivity matrices; restricted (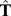) and unrestricted (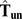). The unrestricted connectivity matrix has been relaxed from any type of topological constraints. In principle, if the network model that was supplied as prior information is indeed close to the real network structure, the elements which were previously restricted to 0 should not deviate far from 0 in the unrestricted connectivity matrix. This information can be accessed via the mean squared sum of these elements, referred to as MSSRE (Mean Squared Sum of Restricted Elements). A similar approach was also used in introducing the Core Consistency Diagnostic (Corcondia) in 3-way analysis [10].

The idea of MSSRE intrinsically assumes that 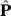 has been properly estimated. This measure would not be appropriate if 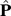 cannot be estimated accurately. A major reason for probable inaccuracy in the estimation of 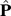 within the NCA framework and how it was challenged will be discussed in more detail later while showing the application of the method on a real biological system.
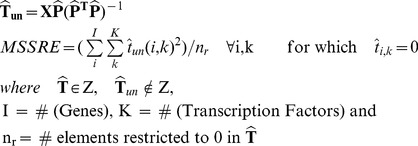
(5)


The unrestricted connectivity matrix gives the opportunity to evaluate the proposed network model from a local point of view as well. Investigation of the individual elements in the unrestricted matrix makes it possible to see which connections in the network model are supported by the gene expression data. Some of the elements which were restricted to 0 in the originally imposed network structure would tend to deviate far from 0 in the unrestricted connectivity matrix more than the others. These elements would indicate additional potential connections in the network. Furthermore, the idea of unrestricted connectivity matrix can be extended to include a new set of genes whose expression profiles were not used for the estimation of 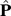.

In addition to the original model set with the I genes in the analysis, the estimated 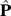 can be used to estimate the connection strengths of the transcription factors with a new the set of L genes which were not previously included in the analysis, 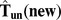 as in Equation 6. For this purpose, the gene expression data of these new genes in the J conditions (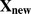) is used. This extension of the approach assumes that 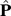 could be properly estimated by using only the expression profiles of the model set genes.
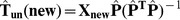
(6)


### Simulations Setup

The main goal of the simulations study was to model the simulated data by embedding different types of prior information during modeling and to elaborate on the measures that made it possible to discriminate between these different cases. In this sense, synthetic data gives us the opportunity to know exactly which network model is closer to the real network structure, 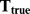 which was used to generate the data. The simulated dataset consisted of 240 genes, 20 transcription factors, 40 different conditions and was constructed based on Equation 7.
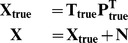
(7)


The values of the elements in the 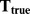, 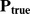 and the measurement noise term, 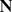 were randomly drawn from standard normal distribution. Then some of the elements in 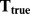 were randomly set to 0 representing the imposed topological pattern. This pattern was very sparse where one gene is regulated by at most 6 transcription factors in order to mimic the sparsity of real biological transcriptional regulatory networks. The level of the added measurement noise was either 0%, 5% or 20%. The noise level was calculated based on the sum of squares of the true expression data, 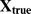 as shown in Equation 8.
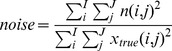
(8)


The simulations were carried out by embedding both correct and incorrect prior information at different noise levels in various transcriptional regulatory network structures. Here, correct prior information (Correct Network (CN) in Table 1) refers to the true regulatory pattern used to create the synthetic network structure as explained earlier. For the incorrect prior information (MN3% and MN5% in Table 1), the connections of randomly selected transcription factors were changed by adding a connection not present and removing another connection which was present in the correct prior information. The properties of the various prior information used in the simulations are shown in Table 1. As shown in Table 1, in a 3% randomly misconnected network structure, 3% of the total number of the connections differ with respect to the correct prior information. We derived 5% randomly misconnected network structures from the 3% misconnected structures by adding extra random misconnections. The misconnection levels were kept low in order to test the discrimination capability of our method even at very low levels. Furthermore, random network structures were generated completely unrelated to the correct one except the size, connectivity and total number of connections in the network. We used random networks to see the changes in the discriminating measures when the prior information is completely incorrect and thus to test whether the discriminating measures relied on chance.

**Table 1:** Prior Information Properties.

Case Label	Type of Prior Information	Remarks
CN	Correct Network	–
MN3%	3% Randomly Misconnected Network	Misconnection level 1 in Figure 3
MN5%	5% Misconnected Network	Misconnection level 2 in Figure 3 -Extra misconnections were added on top of the corresponding readily miswired network structures of case MN3%.-
RN	Random Network	Certain graph properties were kept the same with case CN.

**Figure 3:**
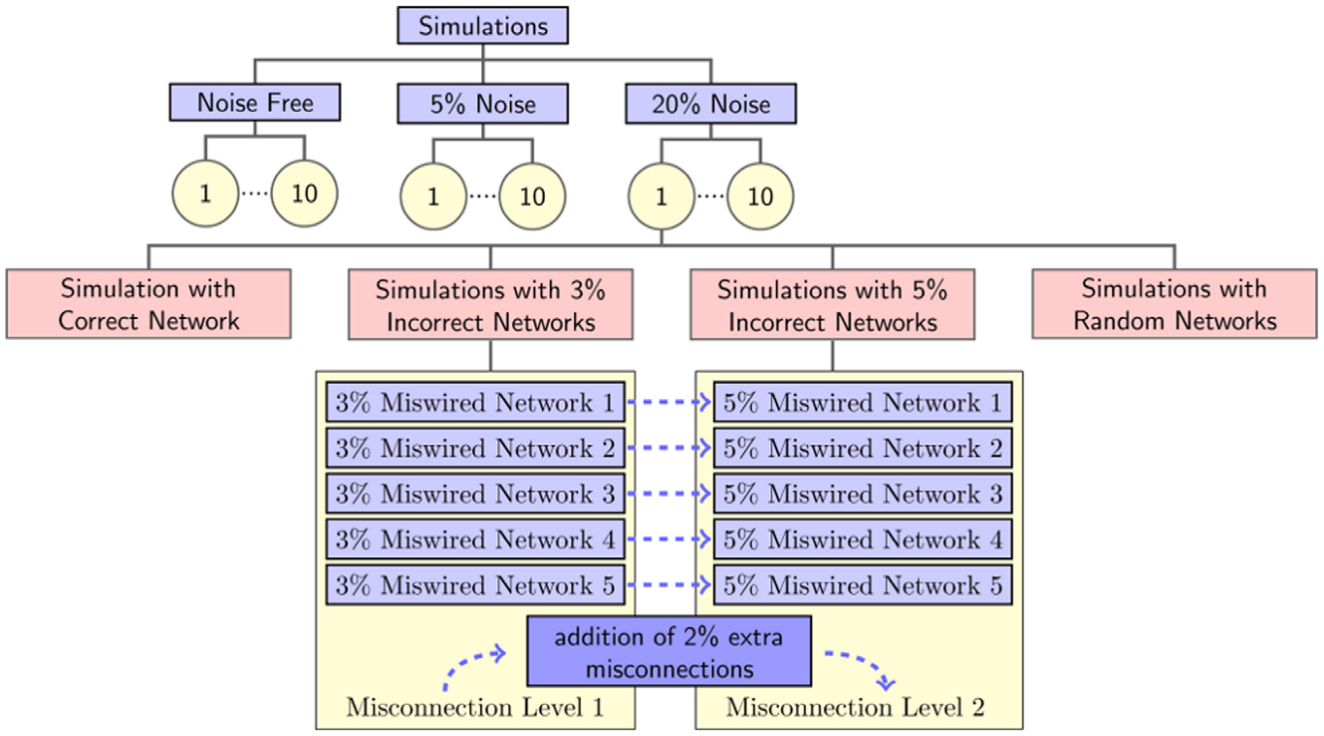
Structure of the Simulations. At each noise level, 10 different regulatory networks were simulated as shown with circles in the figure. Later, each of these were manipulated to obtain different prior information as depicted in pink rectangles.

The structure of the simulations setup is depicted in Figure 3. For each specific regulatory network structure, there existed simulations with four types of prior information (see Table 1 for details). The incorrect prior information cases consisted of 5 different sets of misconnections at each misconnection level as shown in Figure 3 with the yellow boxes. On the other hand, simulations with random prior information were carried out 100 times for each corresponding CN. Most important of all, each of the nodes in this simulation scheme was repeated with 100 different noise realizations. This means that the simulation experiments were repeated 100 times at each case of prior information. This allowed statistical comparison between different cases.

## Results and Discussion

### MSE: A Sensitive Global Measure for Discrimination

**Figure 4:**
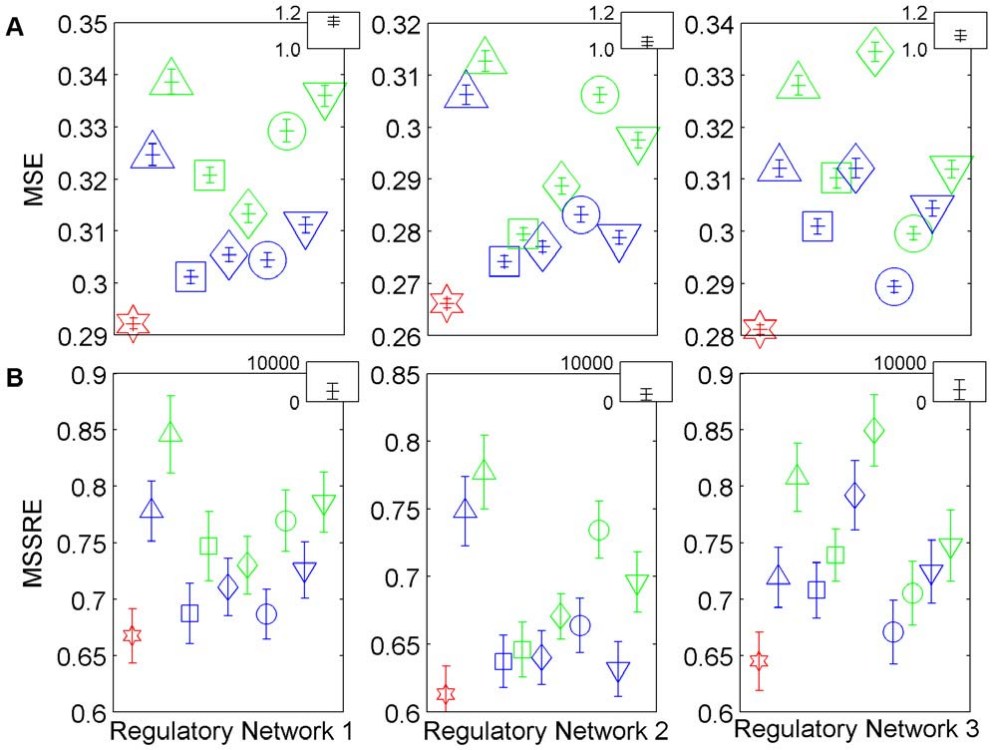
MSE and MSSRE in the simulations with 20% noise. Each point corresponds to the median of the MSE values (**Panel A**) and MSSRE values (**Panel B**) in 100 simulations with different noise realizations. The error bars represent the median absolute deviation. The results of the simulations with the correct network (CN) are plotted in red whereas the blue and green colors represent the solutions with 3% and 5% misconnected network structures, respectively (MN3% and MN5%).The networks which are represented by the green shapes share the same misconnections with the corresponding shapes in blue and have extra misconnections on top of these. In the upper right hand side corner of each plot, the results of the simulations with totally random networks (RN) are depicted. In **Panel A**, each errorbar is surrounded with different shapes, whereas in **Panel B**, the medians are denoted by the corresponding shapes for better readability of both graphs. (See Table 1 for the details of the prior information used).

In Figure 4, the medians and median absolute deviations of MSE are shown for 12 different simulation cases in each of the three example network structures. As can be seen from the figure, the MSE discriminates between different types of prior information steadily well even with 20% noise in the data which was the maximum level of noise in the simulations. We have chosen this noise level based on the expected level of reproducibility in different types of microarray data. In a comprehensive study where they calculated the coefficient of variation of gene expression in replicate experiments, the median of this variation coefficient across all genes changed between 5% and 23% [11]. This indicated that the noise to signal ratio never exceeded 23% in over 80 experiments that they have performed with 6 different platforms. Our limit of 20% thus seems realistic for microarray data in general. The results of simulations with less noise were more apparent so they were not discussed here.

In Figure 4, each shape in blue (MN3%) has an MSE distribution with a higher median than the correct network (CN) model and has an MSE distribution with a lower median than the corresponding same shape in green (MN5%). Besides that, all of these MSE distributions depicted in the main parts of the graphs locate separately from the MSE distributions of totally random network structures (RN) shown in the upper right corners. This shows that when misconnections are introduced in the prior information of network structure, the model fit gets worse. One sided t-tests between these cases with different prior information also indicate that all of these distributions can be separated statistically well from each other at a 5% significance level. This means that the mean of the MSE distribution of the 100 experiments with a MN5% is greater than the mean of both a CN and a MN3%.

What is important here is that the solutions in 100 different noise realizations are very close to each other in each case with a certain prior information. The superiority of using the alternating least squares approach not with random guesses but with an educated initial guess for **T** is important, in this sense because the ordinary least squares optimization with random initial guesses leads to local minima in a considerable number of cases. However, by using an educated initial guess, the local minima problem was encountered only in 0.5% of all the simulations that have been carried out in total. This finding eliminated the need for additional runs with different starting points.

### MSSRE and The Local Investigation of the Unrestricted Network Structure

In accordance with the observations in MSE, the relaxation of topological constraints for calculating the MSSRE led to relatively higher MSSRE in simulations with incorrect prior information (Figure 4). MSSRE acted in a consistent manner with the previously discussed MSE measure. The results of non-parametric tests between these different prior information cases (Figure 4) indicated that the distributions of MSSRE could be separated in 95%of all the comparisons at a 5% significance level. The results suggested that the variability of MSSRE was higher than the MSE. Even when the MSE of different models were small, the values that the originally restricted elements took in 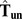 could vary to a higher degree. However, the unrestricted connectivity matrix 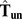 offered more. Inspection of the individual elements in the unrestricted matrix gave indications on the locations of the misconnections. This generates the opportunity for a local evaluation and possible improvement of the proposed network structure.

**Figure 5:**
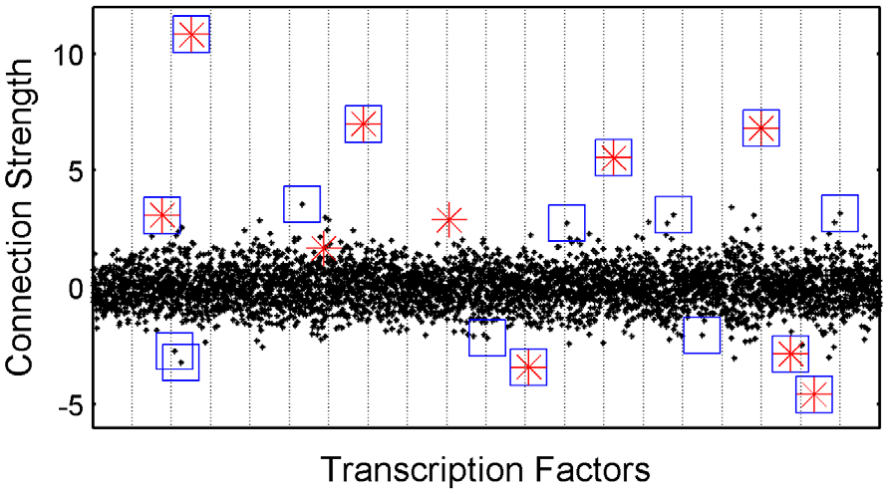
Values of Connection Strengths in 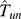. In this plot, each black point represents the connection strength of an element in the unrestricted connectivity matrix which was restricted to 0 previously in the imposed network structure. Each column represents one transcription factor. Red stars are the connections which were missing in the imposed network structure whereas in CN these elements have nonzero values indicating existing connections instead. The outlier elements for each transcription factor identified at a whisker length of 2 are surrounded with additional squares in blue.

In Figure 5, the values of specific elements in the unrestricted connectivity matrix, 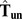 are shown. These specific elements are the ones which were originally restricted to 0 in the proposed network structure. This example figure comes from one of the simulations with MN5% with 20% noise. The connection strengths of these originally restricted elements estimated after the relaxation of the topological constraints are shown with black dots. In some of the elements, the deviation from 0 is very strong and easily distinguishable from the others. These are the outlier connections framed in blue squares.

The outlier connections are the ones that were estimated to be uncommonly strong compared to other connections. Their values were estimated either higher than 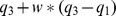 or smaller than 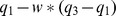, where 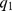 and 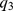 are the 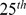 and 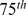 percentiles of the distribution, respectively and w is the whisker length as suggested by [12]. This distribution refers to the distribution of the elements which were originally restricted to 0, in one column of 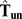 (corresponds to one transcription factor at each time). As a result of their extra-ordinary locations in the very edge of the distributions, they have the potential to point to the existing connections which were missing in the imposed network structure. Therefore, we expect the outlier connections and these missing connections (Figure 5) to overlap at a considerable degree. The non-parametric definition of outliers is very beneficial for our case where the underlying distributions of the connection strengths per transcription factor might not be normal. The sensitivity and false discovery rate for the identification of the missing connections depend on the chosen whisker length (w) as summarized in Table 2. In Table 2, whisker length has been varied from the very loose value, 1.2 up to the extremely strict value of 2.8. When the number of outliers were kept high at a whisker length of 1.2, 72% of the missing connections (denoted by red stars in Figure 5) were identified in the outliers. This indicated the sensitivity of the method. As the whisker length increased, the number of the missing connections which could be identified by the method (true positives) decreased as a result of the decreasing number of outliers detected. This decreased the sensitivity from 0.72 to 0.48. On the other hand, the false discovery rate decreased at a faster rate from 0.95 to even 0.07. When whisker length was set to the most extreme value, only 7% of the outlier connections were false positives. Although it depends on the analyst to decide which one to sacrifice, in most of the cases, we think that the number of false positives should be reduced as much as possible. This performance summary depicted in Figure 2 proves to be a very useful tool when the whisker length has to be optimized for real biological data. The FDR value calculated for simulated data can give good indication of the expected FDR in real data.

**Table 2:** Discrimination Performance.

	Sensitivity	False Discovery Rate
Whisker Length	# Missing connections identified in the outliers/#All missing connections	# False positives in the outliers/# All outliers
1.20	0.7228	0.9487
1.40	0.6897	0.9080
1.60	0.6593	0.8342
1.80	0.6274	0.7114
2.00	0.5980	0.5409
2.20	0.5672	0.3642
2.25	0.5598	0.3231
2.30	0.5528	0.2846
2.40	0.5382	0.2182
2.45	0.5309	0.1885
2.50	0.5237	0.1641
2.60	0.5099	0.1218
2.70	0.4965	0.0909
2.80	0.4830	0.0690

### Overall Results of the Simulations Study

**Figure 6:**
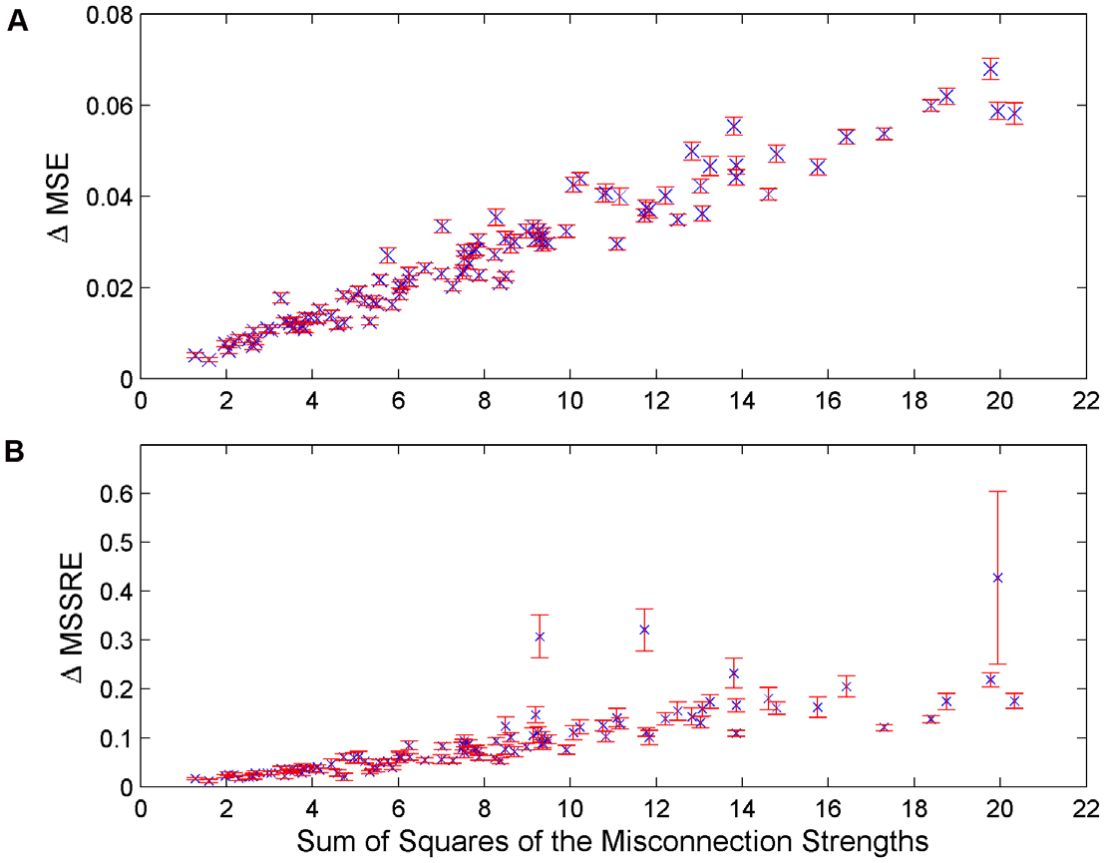
Behaviour of MSE and MSSRE with respect to changes in the connection strengths differing between the competing networks. The change in MSE (Panel A) and MSSRE (Panel B) in simulations with MN3% and MN5% relative to the simulations with CN are shown in the y-axis. In the x-axis, the total magnitude of the missing connections which existed in CN but had been ignored in the imposed network structure are plotted.

For further investigation of the discriminating capacity of the measures, we checked the magnitude of the connection strengths differing between the competing networks. Indeed, the answer to the question whether these networks are easily distinguishable heavily depended on the magnitude of the difference between the competing networks. If the connections which the imposed network (either MN3% or MN5%) lacked were indeed strong connections in the correct network (CN), the differences in both measures with respect to the simulations with CN were shown to be larger. This relation is more clear when Figure 6 is investigated. In these figures, the relative values of the two measures in MN3% and MN5% simulations with respect to their values in CN simulations were shown with respect to the magnitude of the misconnections. The magnitude of the misconnections is formulated as the sum of squares (SS) of all the connection strengths that were existent but later had been restricted to 0 to create the misconnected networks. The differences increased as the Sum of squares of the misconnections increased and thus made the discrimination as clear as it deserves. This conclusion is based on the idea that strong connections deserve more to be identified than the weaker interactions. However, Figure 6 suggests that this dependence on magnitude is weaker in MSSRE. In some cases, the difference in MSSRE was smaller when SS of the misconnections was larger. This can be explained by the dependence of the MSSRE on the topology. Discrimination by MSSRE might be more difficult when certain topologies are involved.

Based on the results we obtained from the simulations study, it can be concluded that MSE and MSSRE both can be used efficiently to discriminate between two competing network structures. The discrimination was possible in 95% of cases with MSSRE and 100% of cases with MSE even when the network structures shared 97% of the connections in common. However, the discrimination capabilities of both measures increased with the strength of the connections differing between the two networks. In this sense, MSE was more successful than MSSRE in discriminating weaker differences. MSSRE seemed to be more dependent on the specific topologies of the networks that were questioned. On the other hand, the unrestricted network structure was worth to be inspected in more detail as a tool for local evaluation rather than global evaluation. When the connection strengths in this unrestricted connectivity matrix were investigated, the unexpectedly outlying elements which deviate far from 0 proved to be mostly the connections which exist in reality but had been ignored in the previously imposed network structure. However this last conclusion must always be carried out with care since the setting of the whisker length for the definition of an outlier heavily affects the sensitivity and false discovery rate in the discrimination process. The values reported for FDR at different whisker lengths in the simulations study can work as a very useful reference for application of the method to real biological systems.

An important point of discussion regarding our method might be the questioning of the applicability range of our method’s local improvement feature. How misconnected can a network be at most to still allow this method to indicate potential connections in the network, and what is the maximum noise level allowing reliable analysis? The actual misconnection level in TF-binding data is thought to be between 10% and 50% [13]. Therefore, we constructed even more misconnected networks (25% misconnection level) to test this particular feature with 30%–50% measurement noise. The sensitivity values were affected by both noise in the gene expression measurements and misconnection level of the network. The sensitivity calculated at the whisker length of 1.2 decreased to 0.54 in the most extreme case with 50% noise and 25% misconnection level. This decrease in the sensitivity at extreme cases indicated that identification of the misconnections became difficult. However, the FDR values were not affected at all by the increasing noise or the misconnection level. The stability of the FDR values calculated at the whisker length of 2.8 (below 0.07) showed that even at these extreme cases, the candidate interactions identified by our approach were very unlikely to include false positives. Keeping the FDR low in such a discovery scheme makes more sense from a biological point of view than achieving high sensitivity. Hence, these results indicated that our local approach is reliable even at these high experimental uncertainties. When the global discrimination was considered, MSE could discriminate between these extreme cases in 100% of the comparisons. The performance of MSSRE in discriminating networks with small differences decreased with increasing noise. However, it could still discriminate in 80% of all the comparisons made between highly similar networks with 95% connections in common. Another important point we observed was that an increase in the misconnection level of the network resulted in an increase in the number of local minima encountered in the simulations. This indicates the need for an optimization scheme with multiple starting points when prior information on the approximate extent of the misconnection levels of the networks is not available. The results of simulations at high experimental uncertainities can be found in Table S1.

**Figure 7:**
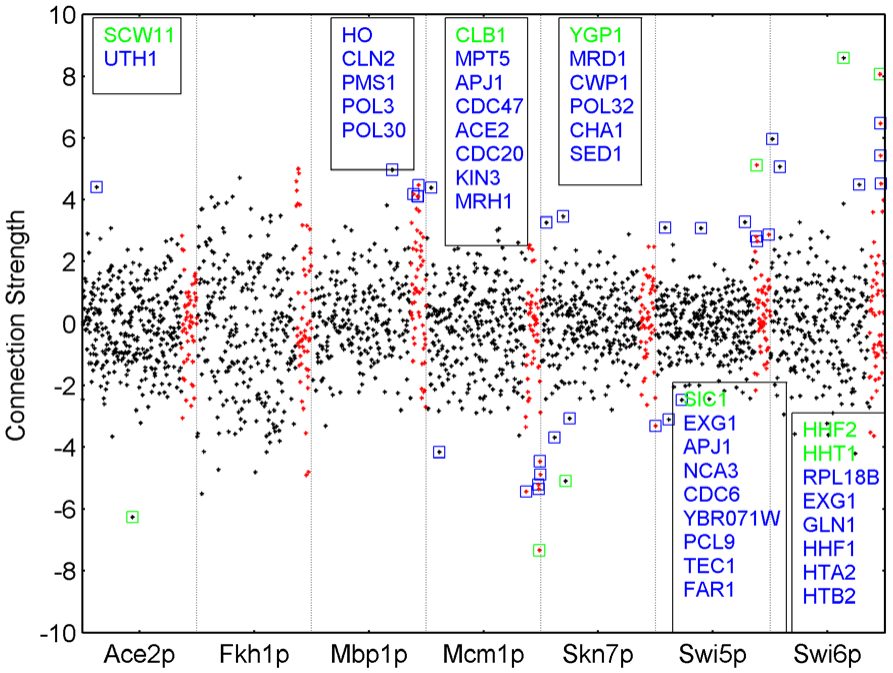
Local Investigation of the Harbison Cell Cycle Transcriptional Regulatory Network. The black dots correspond to the elements in 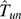 which were restricted to 0 in the imposed Harbison network structure, and the red denotes the elements belonging to the new set genes. Blue squares show the outliers defined at a whisker length of 1.6 and green squares denotes the outliers defined at a whisker length of 2.8. The gene names in these outlier connections are shown in their respective color, as well. In the y-axis, the values of the connection strengths are shown.

### Application to the Cell Cycle Trancriptional Regulatory Network of Yeast

We applied our discrimination algorithm to a real biological transcriptional regulatory network structure. For this purpose, we chose to work on the transcriptional regulatory system controlling the well studied cell cycle in *S. cerevisiae*. The system chosen included 11 cell cycle transcription factors: Ace2p, Fkh1p, Fkh2p, Mbp1p, Mcm1p, Skn7p, Ndd1p, Stb1p, Swi4p, Swi5p and Swi6p. For the identifiability of the network, Ndd1p had to be removed [2]. The network structure regarding these transcription factors was adapted from the study of Harbison *et al.* [14], named the Harbison network throughout the text. In this benchmark study, the genes that are likely to be targets for transcriptional regulators have been identified by consensus of information from genome-wide location data, phylogenetically conserved sequences and prior information. For our purposes, we used the most reliable transcription factor-gene interactions with a binding p-value smaller than 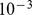 that have been conserved in at least two yeast species.

Cell cycle microarray data from Spellman *et al.* was used for the analysis [15]. The data analyzed consisted of time series gene expression data from four synchronization experiments, leading to a total of 77 sampled conditions.

**Table 3:** Predicted Interactions in Cell Cycle Regulatory Network of Yeast.

Whisker Length	TF	Gene	Biological Evidence
2.8	Ace2p	*SCW11*	ChIP [17], [19]
	Mcm1p	***CLB1***	ChIP [19], [20], Gene is known to be regulated in the G2/M phase of the cell cycle [21], Mcm1p is an important transcriptional regulator of this phase [17].
	Swi5p	***SIC1***	Regulation of *SIC1* gene by the Swi5p was already known [17], [22].
	Swi6p	*HHF2*, ***HHT1***	These histone genes (together with their homologues *HHF1* and *HHT2*) were shown to be both MBF (Mbp1p-Swi6p complex) and SBF (Swi4p-Swi6p complex) targets [23].
1.6	Ace2p	*UTH1*	ChIP [19]
	Mbp1p	*RAD27*	ChIP [17], [19]
	Mbp1p	*CWP1*	Comparative Microarray [24]
	Swi5p	*EXG1*, *CLN3*	ChIP [17], [19]
	Swi5p	*CDC6*	Gene’s transcription at the end of mitosis is induced by Swi5p [25].
	Swi6p	*RPL18B*, *EXG1*	ChIP [17], [19]
	Mbp1p	***POL3***, ***POL30***, ***PMS1***	Identified as late G1 phase genes regulated by MBF complex [26], [27].
	Swi5	***PCL9***	It is known that the expression of the gene is regulated by Swi5p [28].
	Swi5	***TEC1***	ChIP [17]
	Mcm1p	***ACE2***, ***CDC20***, ***KIN3***, ***MRH1***, ***CDC47***	ChIP [20], Genes are regulated mainly in G2/M phase [15], [21], [29]–[31] or M/G1 boundary (*CDC47*) [32] and Mcm1p is known to be involved in the transcriptional regulation of these phases either by direct binding or together with Fkh1p, Fkh2p and Ndd1p [17].
	Swi6p	***HTA2***, ***HTB2***, ***HHF1***	These histone genes are targets for both MBF (Mbp1p-Swi6p complex) and SBF (Swi4p-Swi6p complex) [17], [23].

Only the ones which were supported by biological evidence from literature are shown here. **Bold font** genes belong to the new set of genes whereas the normal font genes are the model set genes.

#### Problem of degeneracy

A degeneracy problem arose when the expression data was modeled with 10 underlying factors. This led to an extremely high condition number of the estimated activity matrix which indicated that the activities of different transcription factors were linearly dependent on each other. However, for proper discrimination between networks independent profiles are required, because otherwise the activity profiles and thus the connections of the transcription factors cannot be distinguished. It is very important to notice that, in such situations, one of the criteria for identifiability [2] is severely disturbed. This loss in the rank of the activity matrix might be easily missed due to the compensation by the noise in the system. In other words, noise in the data may hide the elevated levels of linear dependence between the activity profiles of the transcription factors. In the end of the analysis, estimated profiles of the transcription factors might be extremely correlated although there is no loss in the calculated rank of the activity profile matrix.

When more factors are extracted from a data set than can be supported by it, this kind of degeneracy occurs [16]. The SVD decomposition of the data also indicated clearly that the data should be modeled with fewer underlying factors. A scree plot of the singular values revealed an optimal number of 7 independent factors. This high dependency between the activity profiles can be explained by the partial redundancy and serial regulation structure regarding different cell cycle regulators. Indeed, in earlier studies it was shown that cell cycle regulators had important roles in controlling each other’s expression profiles [17]. A high degree of overlap between the target genes of cell cycle regulators was also mentioned in the same study. These regulators were not only homologues or partners in regulating complexes, but they could also be regulators that were not known to be related at all in terms of their specific role in regulation. These findings support our estimated dependency between the factors.

To find out the best combination of 7 transcription factors, a trade-off approach was used. Out of all the possible combinations, a set of factors was selected that were most independent but yet resulted in low residuals and thus good models of the data. For this aim, decompositions were carried out several times with all possible combinations of 7 transcription factors. Among the models with the best fit, we looked for the smallest condition number of 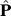. This set of transcription factors both described the data well, indicated by the low residuals of the model, and were independent of each other, indicated by the small condition number of 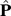. The resulting high confidence network contained the interactions between 342 genes and the 7 transcription factors: Ace2p, Fkh1p, Mbp1p, Mcm1p, Skn7p, Swi5p and Swi6p.

Another general solution to this degeneracy problem would be increasing the number of experimental conditions. At these new data points, the connectivity structure of the network should be the same but the biological interdependency of transcription factors should be lower. That would allow independent activity profiles in 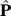 but it also necessarily requires design of new experiments and specific selection of data points. Indeed in [3], the authors have followed a specific application of such an approach where they incorporated microarray data from transcription factor deletion mutants. They achieved this incorporation by putting constraints on 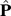 such as zeros for certain elements. As a conclusion, carrying out new microarray experiments might solve the problem of degeneracy while a purely computational solution remains as a challenge.

#### Two competing networks for cell cycle regulation

We compared the Harbison network [14] to another network that has been constructed by reanalyzing the same ChIP data [13] by MacIsaac *et al.* This alternative network has been reported as an improved map of the regulatory network in *S. cerevisiae*. We only included the genes and the transcription factors that have reported interactions in both networks. When the number of transcription factors was further reduced to overcome the non-identifiability and degeneracy problems, both networks included 308 genes and 7 transcription factors. The MSE values for the two networks did not differ significantly (0.1312 and 0.1313, respectively). This suggests that the cell cycle related part of the MacIsaac regulatory network used for this study does not show significant improvement in comparison to the Harbison network. It is important to note here that the size of the networks that can be compared is severely limited by the limitations of the NCA approach. First of all, the networks both must be identifiable, as has been discussed by Liao *et al.* in [2]. Secondly, the transcription factors involved in the study must have independent activity profiles as we have already discussed. Due to these restrictions, we could only compare certain parts of the cell cycle regulatory network. Still, we showed that this part represents the whole cell cycle regulatory mechanism well. The details regarding this latter assessment was already discussed in the previous section where we discussed about the best combination of the transcription factors. Another reason behind the insignificant improvement might be due to the connection strengths of the connections differing between the two networks. As we have already discussed in the results section for the simulations, networks with strong connections differing are more easily discriminated than the ones with weaker connections differing. It might be the case that the regulatory interaction map achived in [13] is indeed more realistic but these interactions that exist in the part of the network we tested are not strong enough to be identified. However, local investigation of the Harbison network showed possible points of improvement in the network as will be discussed in the next section.

#### Emerging interactions

The unrestricted Harbison cell cycle transcriptional regulatory network, 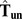 was calculated according to Equation 9 with an extension to a new set of 54 genes. The idea behind was introduced in Equation 6. The expression values of this new set of genes, 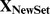 were not taken into account for estimating 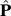 but were used for estimating their connection strengths with the transcription factors in the study as described before for simulated data. The genes in the new set were known to be cell cycle regulated [15] but had not been included in our network. The reason was that these genes were not connected to any of the transcription factors based on the interaction data adapted from [14] and thus were previously excluded from the analysis. 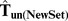 stored the unrestricted connection strengths of the 54 new set genes whereas 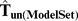 stored the unrestricted connection strengths of the 342 model set genes whose expression values were used to estimate 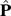.

Equation 9 also shows clearly how the degeneracy problem would lead to difficulties associated with the identification of the new interactions in both the model set and the new set. High condition number of 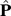 would make it nearly rank deficient. This would introduce errors in the generalized inverse of 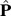 used in Equation 9 and thus also in the 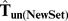 and 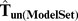.
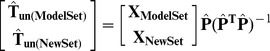
(9)


Figure 7 shows the connection strengths estimated in 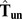. For each transcription factor, outlier connections were identified as described earlier for simulated data. The elements surrounded with squares are of high importance because they potentially point to connections which indeed do exist but had not been included in the network structure used. In the same figure, the boxes in each segmentation show the gene names for these potential interactions of each transcription factor.

When the whisker length was kept at 2.8, only 6 outliers were detected. Based on the performance evaluation in Table 2 we expect nearly 0 false positives in this set. It must not be forgotten that the real biological data will show differences in terms of sensitivity and false discovery rate compared to the simulated data. However, the performance measures for simulated data can still give an approximate idea of the discrimination performance in real data. This was also supported by the findings in real data when the outlier elements were further investigated. There is strong biological evidence for 5 out of the 6 outliers pointing to existing regulatory interactions between genes and transcription factors (Table 3). In such a case, the remaining predicted interaction (Skn7p with the *YGP1* gene) is worth being investigated further both through literature and experimentation.

The whisker length can be reduced to let more outliers show up in the analysis. This will identify weaker interactions at the cost of a higher incidence of false positives. We set the whisker length to a relatively loose value of 1.6, to let the number of outliers increase to 38. Out of these 38 potential interactions, nearly half come from the new set of genes, as expected. The new genes were curated from literature as cell cycle regulated genes but had no interactions according to the imposed Harbison network structure. Therefore their regulation pattern was non-existent in the network used as prior information, and they immediately showed their regulation pattern in the unrestricted connectivity matrix.

Out of the model set connections, evidence for 10 of them was found in other sources of experimental data (Table 3). When the new set was considered the number of connections supported with biological evidence increased to 17 (Table 3).

Lastly, there are a considerable number of outliers that can be hypothesized regarding the Skn7p. Apparently, the activity profile of this transcription factor is essentially needed to explain the expression profile of these genes. However, we know from literature that Skn7p’s role in cell cycle regulation is through its association with the Mbp1p [18], but there is little information about this role in the literature which is why we choose not to interpret these interactions in more detail here.

### Conclusions

We present this study as a contribution to both model discrimination and model improvement in the rapidly evolving world of network based approaches. In terms of model discrimination, we have presented measures to discriminate between competing regulatory network structures. Looking at the MSE and MSSRE in decompositions with two different network structures allows to comment on the consistency between the data and the network structure. This indicates which network structure is the most realistic. However, the magnitudes of differences in these measures between two networks depend on the total strength of the connections differing between them. It is therefore easier to discriminate between networks with strong connections differing between one another. This conclusion makes sense through biological interpretation: strong connections are more easily identifiable and they should be so. This finding is more consistent in MSE whereas MSSRE is more dependent on the specific topologies that are questioned. Therefore, we suggest MSE as a sensitive global measure that discriminates between two different networks.

In terms of model improvement, the relaxation of the topological constraints for the estimation of an unrestricted connectivity matrix allows us to investigate the connections individually. Through this local approach, the unexpectedly strong connections in the unrestricted connectivity matrix can be identified as outliers. These outliers point to existing connections that were lacking in the hypothesized network as has been shown on simulated data. We also showed how the application of the method to the cell cycle regulatory network of *S. cerevisiae* led to the prediction of novel regulatory interactions, improving the information content of the hypothesized network model.

## Supporting Information

Table S1 **Summary of the simulations with high experimental uncertainities.** The sensitivity and FDR values regarding the local part of our approach are given at two different whisker lengths. PMSE and PMSSRE stand for the percentage of the simulations where global measures MSE and MSSRE could discriminate between different networks, respectively. The misconnection levels of the networks that have been compared are stated in the second column.
(PDF)
