# Supplementary material for: Topology of Transcriptional Regulatory Networks: Testing and Improving
Source: PLoS One. 2012 Jul 23;7(7):e40082. doi: 10.1371/journal.pone.0040082 (PMC3402518; doi:10.1371/journal.pone.0040082)
Supplement: Table S1 — Summary of the simulations with high experimental uncertainities. The sensitivity and FDR values regarding the local part of our approach are given at two different whisker lengths. PMSE and PMSSRE stand for the percentage of the simulations where global measures MSE and MSSRE could discriminate between different networks, respectively. The misconnection levels of the networks that have been compared are stated in the second column. (PDF) [file pone.0040082.s001.pdf]

Supplementary Information

Table S 1. Summary of the simulations with high experimental uncertainities. The sensitivity and FDR values regarding the local part of our approach are given at two different whisker lengths. P_{MSE} and P_{MSSRE} stand for the percentage of the simulations where global measures MSE and MSSRE could discriminate between different networks, respectively. The misconnection levels of the networks that have been compared are stated in the second column.

		Whisker Length = 1.2		Whisker Length = 2.8			
Noise Level	Misconnection Level	False Discovery Rate	Sensitivity	False Discovery Rate	Sensitivity	P_{MSE}	P_{MSSRE}
30 %	3% & 5%	0.96	0.66	0.06	0.40	100 %	72 %
	10% & 20%	0.85	0.66	0.06	0.38	100 %	100 %
	20% & 25%	0.79	0.63	0.07	0.34	100 %	82 %
40 %	3% & 5%	0.96	0.61	0.06	0.33	100 %	62 %
	10% & 20%	0.86	0.61	0.04	0.31	100 %	100 %
	20% & 25%	0.80	0.58	0.06	0.28	100 %	82 %
50 %	3% & 5%	0.95	0.57	0.06	0.27	100 %	56 %
	10% & 20%	0.86	0.57	0.04	0.26	100 %	88 %
	20% & 25%	0.81	0.54	0.05	0.23	100 %	80 %
